# Enzymes involved in DNA ligation and end-healing in the radioresistant bacterium *Deinococcus radiodurans*

**DOI:** 10.1186/1471-2199-8-69

**Published:** 2007-08-16

**Authors:** Melanie Blasius, Rebecca Buob, Igor V Shevelev, Ulrich Hubscher

**Affiliations:** 1 Institute of Veterinary Biochemistry and Molecular Biology, University of Zürich-Irchel, Winterthurerstrasse 190, 8057 Zürich, Switzerland; 2 Donnelly Centre for Cellular and Biomolecular Research (CCBR), Department of Biochemistry & Department of Medical Genetics and Microbiology University of Toronto, 160 College Street, Toronto, Canada

## Abstract

**Background:**

Enzymes involved in DNA metabolic events of the highly radioresistant bacterium *Deinococcus radiodurans *are currently examined to understand the mechanisms that protect and repair the *Deinococcus radiodurans *genome after extremely high doses of γ-irradiation. Although several *Deinococcus radiodurans *DNA repair enzymes have been characterised, no biochemical data is available for DNA ligation and DNA endhealing enzymes of *Deinococcus radiodurans *so far. DNA ligases are necessary to seal broken DNA backbones during replication, repair and recombination. In addition, ionizing radiation frequently leaves DNA strand-breaks that are not feasible for ligation and thus require end-healing by a 5'-polynucleotide kinase or a 3'-phosphatase. We expect that DNA ligases and end-processing enzymes play an important role in *Deinococcus radiodurans *DNA strand-break repair.

**Results:**

In this report, we describe the cloning and expression of a *Deinococcus radiodurans *DNA ligase in *Escherichia coli*. This enzyme efficiently catalyses DNA ligation in the presence of Mn(II) and NAD^+ ^as cofactors and lysine 128 was found to be essential for its activity. We have also analysed a predicted second DNA ligase from *Deinococcus radiodurans *that is part of a putative DNA repair operon and shows sequence similarity to known ATP-dependent DNA ligases. We show that this enzyme possesses an adenylyltransferase activity using ATP, but is not functional as a DNA ligase by itself. Furthermore, we identified a 5'-polynucleotide kinase similar to human polynucleotide kinase that probably prepares DNA termini for subsequent ligation.

**Conclusion:**

*Deinococcus radiodurans *contains a standard bacterial DNA ligase that uses NAD^+ ^as a cofactor. Its enzymatic properties are similar to *E. coli *DNA ligase except for its preference for Mn(II) as a metal cofactor. The function of a putative second DNA ligase remains unclear, but its adenylyltransferase activity classifies it as a member of the nucleotidyltransferase family. Characterization of another protein from the same operon revealed a 5'-polynucleotide kinase with a possible role in DNA strand-break repair.

## Background

### *Deinococcus radiodurans*

*Deinococcus radiodurans (D. radiodurans) *exhibits an extraordinary resistance to ionizing radiation. Ionizing radiation generates a variety of DNA damages, including many types of base damages as well as single-strand and double-strand breaks, the latter being the most lethal damage for a living cell. *D. radiodurans *can survive irradiation up to 5,000 Gy without measurable loss of viability, and it seems likely that this resistance is based on mechanisms that ensure limited DNA and protein degradation and provide an efficient and accurate DNA strand-break repair [1]. High intracellular levels of Mn(II) protect proteins and allow fast repair of damaged DNA after irradiation [2,3]. Prokaryotes can repair double-strand breaks by homologous recombination, but proteins implicated in non-homologous end-joining have also been identified recently, such as Ku homologs and additional DNA ligases [4,5]. However, no Ku homolog has been discovered in the genome of *D. radiodurans*. Zahradka *et al*. found that a mechanism called extended synthesis-dependent strand-annealing accounts for most of the strand-break repair [6], although additional DNA repair pathways might contribute to the efficient DNA repair. In any case, a DNA ligase is essential for DNA repair and a 5'-polynucleotide kinase/3'-phosphatase would ensure that DNA strand-breaks could be invariably ligated.

### DNA ligases

DNA ligases play essential roles in replication, recombination and repair since they join broken DNA strands by catalysing the formation of a phosphodiester bond between the 3' hydroxyl end of one strand and the 5' phosphate end of another. Ligation occurs via three nucleotidyltransfer steps: (i) a covalent enzyme-adenylate intermediate is formed, (ii) the adenylate group (AMP) is transferred to the 5'-phosphate terminus of the DNA molecule and (iii) the gap in the DNA molecule is sealed when the DNA ligase catalyses displacement of the AMP residue through the attack by the adjacent 3' hydroxyl group of the DNA [7]. For all DNA ligases, the AMP is linked to a highly conserved lysine residue in the catalytic motif of the enzyme. DNA ligases can use either ATP or NAD^+ ^as an AMP-donor. NAD^+^-dependent DNA ligases are found exclusively in bacteria, certain archaea, and viruses whereas ATP-dependent DNA ligases can be found in eukaryotes, archaea and several viruses including bacteriophages. Recently, it was shown that some bacterial genomes also encode an additional ATP-dependent DNA ligase, some of which were further characterised [7].

The *D. radiodurans *genome contains the gene DR2069 encoding an NAD^+^-dependent DNA ligase, here designated as LigA. The gene DRB0100 encodes another possible diverged homolog of ATP-dependent ligases. As the function of this protein remains unclear it will be called DRB0100 throughout this paper. This predicted ATP-dependent DNA ligase contains all catalytic residues, and its expression is strongly upregulated upon γ-irradiation [8]. In addition, DRB0100 belongs to a putative DNA repair operon together with the genes DRB0098 and DRB0099. DRB0098 has been predicted to encode a kinase/phosphatase with an unusual domain architecture [9] whereas DRB0099 is classified as a domain of unknown function with weak similarity to the macro domain family [10].

### Polynucleotide kinases and 3' phosphatases

Not all DNA strand breaks possess ligatable ends, i.e. a 5' phosphate and a 3' OH terminus. The 5' phosphate can be missing and γ-irradiation and reactive oxygen can lead to the formation of 3' phosphate or phosphoglycolate ends [11,12]. Enzymatic activity is required to remove the 3' phosphate moiety and to phosphorylate the 5' end at the DNA nick to allow for DNA ligation. Both reactions are catalysed by bifunctional PNKPs. The best characterised PNKP is T4 PNK that is involved in the repair of host tRNA [13]. Additional PNKPs were identified in other viruses and all these viral enzymes can use either DNA or RNA as a substrate. PNKPs were also found in some eukaryotes, e.g. human, *Caenorhabditis elegans *and *Schizosaccharomyces pombe*, where they seem to play an important role in the repair of single-strand and double-strand breaks [14-16]. However, the eukaryotic enzymes can only use DNA as a substrate. Pnk1 from *Schizosaccharomyces pombe *possesses both 3'-phosphatase and 5'-polynucleotide kinase activities, whereas TPP1 from *Saccharomyces cerevisiae *shows only 3'-phosphatase activity. In other organisms, the kinase and phosphatase activities seem to be uncoupled as well, e.g. in *Arabidopsis thaliana*. Only one bacterial PNKP from *Clostridium thermocellum *has been characterised so far [17], showing similarity to viral PNKPs. *D. radiodurans *also seems to possess a PNKP encoded by the gene DRB0098, although the PNKP possesses a special domain architecture [9]. The order of the phosphatase and kinase domains is the similar to eukaryotic PNKPs; in contrast, viral PNKPs have a reversed order of the two domains. The predicted phosphatase domain of the *D. radiodurans *PNKP belongs to the HD hydrolase superfamily [18], and, so far, only one viral PNKP containing this domain has been shown to possess 3'-phosphatase activity [19]. The *D. radiodurans *PNKP is part of the putative DNA repair operon together with the predicted ATP-dependent DNA ligase DRB0100 and the expression of this operon is strongly upregulated upon irradiation. Thus, a role for the encoded proteins in DNA repair has been suggested [8].

In this work we analyse two putative DNA ligases and one predicted 5'-polynucleotide kinase/3'-phosphatase from *Deinococcus radiodurans*.

## Results

### Prediction of two DNA ligases for *D. radiodurans*

**Figure 1:**
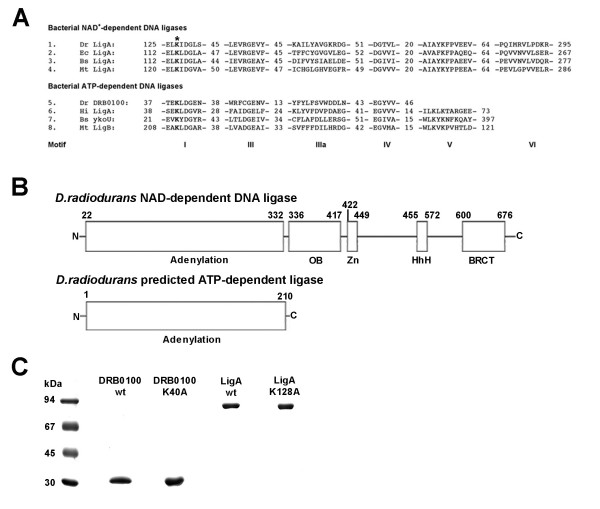
**Alignment and purification of two predicted *D. radiodurans *DNA ligases**. A. Alignment of eight colinear sequence elements in bacterial DNA ligases based on previous studies of DNA ligase motifs [7, 34] using CLUSTALW alignment [35]. The numbers of amino acids between the motifs are indicated. The alignment of motif VI is not shown for the ATP-dependent DNA ligases since the homology is very poor. Note that the putative ATP-dependent DNA ligase from *D. radiodurans *seems to lack also motif V. The conserved adenylated lysine residue is depicted in bold and labelled with an asterisk. Dr, *Deinococcus radiodurans*, Ec, *Escherichia coli*, Bs, *Bacillus subtilis*, Mt, *Mycobacterium tuberculosis*, Hi, *Haemophilus influenzae*. B. Predicted domain structures of *D. radiodurans *NAD^+^-dependent DNA ligase (LigA) and ATP-dependent DNA ligase (DRB0100). The LigA protein scheme is based on homology searches using the NCBI conserved domain database and the SMART conserved domain database. OB, oligonucleotide-binding fold, Zn, zinc finger, HhH, helixhairpin-helix motif 1, BRCT, BRCA1 C-terminal domain. C. LigA and DRB0100 and their corresponding adenylation mutants LigA K128A and DRB0100 K40A were purified over one metal affinity column and two ion exchange columns to near homogeneity as described in Methods. 3 μg of each protein were loaded onto a 10% SDS-PAGE and the gel was stained with Coomassie Blue R250.

Sequence comparison of the two predicted *D. radiodurans *DNA ligases with other bacterial DNA ligases showed that LigA displays a strong similarity to other NAD^+^-dependent DNA ligases (Figure 1A) and comprises the expected adenylation, OB fold and BRCT domains. Like other NAD^+^-dependent DNA ligases LigA also contains a zinc finger and a helix-hairpin-helix motif presumably involved in DNA binding (Figure 1B). By contrast, the predicted ATP-dependent DNA ligase DRB0100 shows poor sequence similarity to other bacterial ATP-dependent ligases, but contains all catalytic residues (Figure 1A and [8]). The DRB0100 protein consists of the adenylation domain only and lacks all other domains present in LigA (Figure 1B); especially no DNA binding motif could be detected.

### Purification of two recombinant DNA ligases from *D. radiodurans*

Both genes encoding putative DNA ligases, DRB0100 and DR2069, were amplified from genomic *D. radiodurans *DNA using specific primers (see Table 1) and cloned into a pRSETb vector for recombinant protein expression in *E. coli *cells with a hexahistidine tag at the N terminus. For both proteins, adenylation mutants were created by replacing the conserved lysine residue with an alanine, resulting in a DRB0100 K40A mutant and a LigA K128A mutant, respectively. All wild-type and mutant proteins were expressed in *E. coli *BL21(DE3) cells and purified to near homogeneity over a HisTrap™ HP column and two additional ion exchange columns (Figure 1C).

**Table 1:** PCR primer sequences used in this study

**Primer name**	**Used for**	**Sequence (5'-3')**
DRB0100F	cloning of DRB0100wt into pRSETb	CGC*GGATCC*GATGCGAGTCAAATACCCTTC
DRB0100R	cloning of DRB0100wt into pRSETb	CGC*GGATCC*GTCATGACTGCTCCTGGCG
DRB0100_mutF	introduction of K40A mutation into DRB0100	CGTCGTGACCGAG**GC**GCTCGACGGCG
DRB0100_mutR	introduction of K40A mutation into DRB0100	CGCCGTCGAGC**GC**CTCGGTCACGACG
DR2069F	cloning of DR2069 wt into pRSETb	CGC*GGATCC*GATGCGTTACCCTGGGCGC
DR2069R	cloning of DR2069 wt into pRSETb	CGC*GGATCC*GTCAGCTTTCAGCGGGGGC
mut_DR2069F	introduction of K128A mutation into DR2069	CCGGCGAGCTG**GC**AATCGACGGCCT
mut_DR2069R	introduction of K128A mutation into DR2069	CAGGCCGTCGATT**GC**CAGCTCGCCGG
DRB0098F	cloning of DRB0098 into pRSETb	CGC*GGATCC*GATGAACCGCAAAAACCGTAC
DRB0098R	cloning of DRB0098 into pRSETb	CGC*GGATCC*GTCAGGAGGTAGATGAGGGCAG
98_R371LF	introduction of R371K mutation into DRB0098	GGTCAGCTCGGAGCA**AAA**ATCAGCGGGAGAGAGC
98_R371LR	introduction of R371K mutation into DRB0098	GCTCTCTCCCGCTGAT**TTT**TGCTCCGAGCTGACC

All oligonucleotides were desalted and used in a final concentration of 0.4 μM. T7 sequencing primers can be found at the Microsynth webpage. Bold bases represent those exchanged in the site-directed mutagenesis. Restriction sites are shown in italics.

### A DNA ligase from *D. radiodurans *performs efficient strand joining in the presence of NAD^+ ^and Mn(II) and possesses adenylyltransferase activity

**Figure 2:**
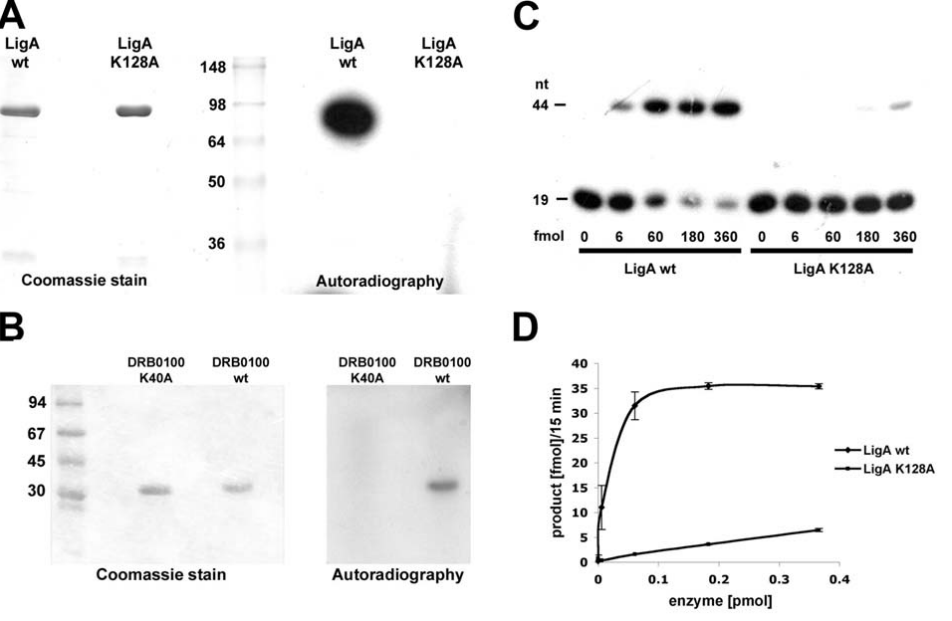
**DNA ligation and adenylyltransferase activities of the putative recombinant DNA ligases**. A. LigA wt and LigA K128A were incubated with [^32^P]-NAD^+ ^and adenylyltransferase activity was detected by SDS-PAGE. Protein bands were visualized with Coomassie Blue R250 (left) and by autoradiography (right). B. DRB0100 wt and K40A were incubated with α-[^32^P]-ATP. Protein-AMP complexes and free α-[^32^P]-ATP were separated by SDS-PAGE. Proteins were stained with Coomassie Blue R250 (left) and detected by autoradiography (right). C. Titration of LigA wt and LigA K128A on a nicked DNA substrate. Indicated amounts of LigA wt and LigA K128A were incubated with the DNA substrate as described in the Methods section. [^32^P]-labelled DNA oligonucleotides were visualized by autoradiography. D. Quantification of three independent experiments as shown in C. Error bars are given as the standard error of the mean.

We tested the ability of the LigA wt and the K128A mutant to ligate a duplex DNA substrate containing a single nick. Ligase activity was measured as conversion of a 5'-[^32^P]-labelled deoxyribose oligomer of 19 nucleotides into an internally labelled oligomer of 44 nucleotides. LigA showed maximum ligation activity with 1 mM MnCl_2_, 5 μM NAD^+ ^and a pH of 6.8 at a temperature of 30°C. Higher concentrations of MnCl_2 _or NAD^+^ had an inhibitory effect on the enzymatic activity. The enzyme was 10 times less active in the presence of MgCl_2_, and even inactive when tested with 1 mM ATP (data not shown). To exclude the possibility that the observed activity is caused by a copurified *E. coli *ligase, we created a K128A mutant that lacks the proposed site of adenylation (Figure 1A). The LigA K128A mutant showed almost no ligation activity confirming that the observed ligation activity results from the *D. radiodurans *NAD^+^-dependent DNA ligase (Figure 2A). The residual DNA ligation does probably not result from a contamination with *E. coli *DNA ligase, as the activity was strongly decreased in presence of 4 mM MgCl_2_, which is optimal for *E. coli *DNA ligase (data not shown). In an adenylyltransferase activity assay LigA wt formed an AMP-ligase complex, whereas complex formation was not detected with the K128A mutant (Figure 2A, right). Thus, lysine 128 is essential for the first step of DNA ligation. The kinetic analysis of the wt reaction using different concentrations of nicked DNA displayed typical Michaelis-Menten kinetics with an apparent *K*_M _of 105 ± 16 nM (Table 2).

**Table 2:** *K*_M _values of prokaryotic NAD^+^-dependent DNA ligases

**Organism**	**T [°C]**	***K*_**M **_[nM]**	**Reference**
*D. radiodurans*	30	105 ± 16	This work
*E. coli*	18	179	Georlette *et al*., 2000
*E. coli*	30	702	Georlette *et al*., 2000
*E. coli*	45	2040	Georlette *et al*., 2000
*P. haloplanktis*	4	165	Georlette *et al*., 2000
*P. haloplanktis*	18	296	Georlette *et al*., 2000
*P. haloplanktis*	25	631	Georlette *et al*., 2000
*T. scotoductus*	45	236	Georlette *et al*., 2000
*T. scotoductus*	60	465	Georlette *et al*., 2000

*K*_M _values for nicked DNA substrates. Details for *K*_M _determination of *D. radiodurans *NAD^+^-dependent DNA ligase are described in Methods. *K*_M _is the mean of 3 independent experiments and the error is given as standard error of the mean.

### Divalent cation dependence and specificity of the DNA strand-joining by LigA

**Figure 3:**
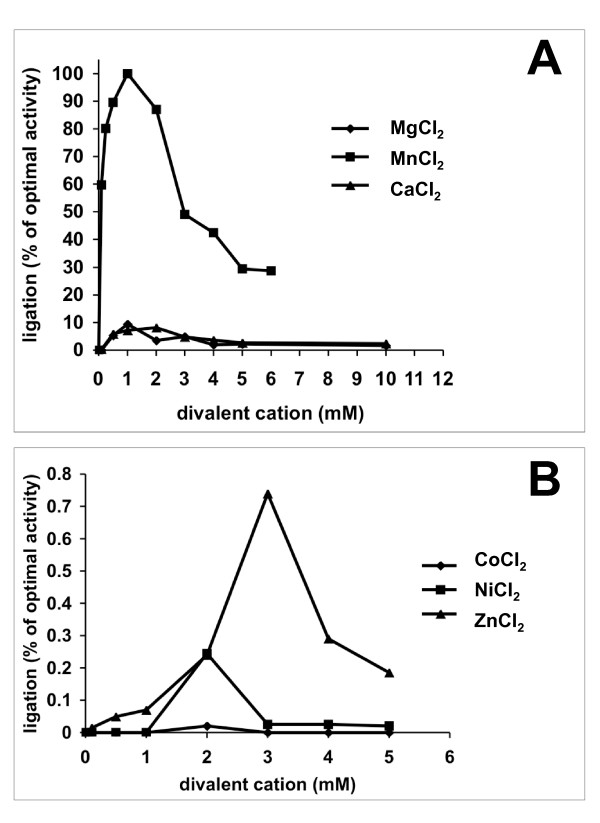
**Divalent cation requirements for LigA activity**. A. Titration of MgCl_2_, MnCl_2 _and CaCl_2_. Ligation assays were performed with 60 fmol of LigA wt and increasing amounts of divalent cations, and quantified as described in Methods. Ligation activity obtained with 1 mM MnCl_2 _was set as 100% and relative DNA ligation activity is shown as the average of 2 experiments. B. Titration of CoCl_2_, NiCl_2 _and ZnCl_2_. Ligation assays were performed as in A. Note that the ordinate has a scale of about 2 orders of magnitude lower than in A for better illustration of the divalent cation optima.

Ligation of a nicked DNA by LigA required a divalent cation cofactor and was best in the presence of 1 mM MnCl_2 _(Figure 3A). MnCl_2 _could be replaced by MgCl_2 _or CaCl_2 _leading however to a 10-fold decrease of activity (Figure 3A). The optimal concentration of divalent cation was 1 mM for MgCl_2 _and 2 mM for CaCl_2_. Only low levels of DNA ligation were observed with NiCl_2_ and ZnCl_2_, the optimal concentrations being 2 and 3 mM, respectively (Figure 3B). CoCl_2 _could not serve as a divalent cation cofactor (Figure 3B).

### DRB0100, a predicted ATP-dependent DNA ligase from *D. radiodurans*, forms a complex with AMP, but does not ligate DNA or RNA *in vitro*

DRB0100 has been predicted to be an ATP-dependent DNA ligase consisting only of the adenylation domain. We first tested whether the DRB0100 protein possesses an adenylation activity using ATP as an AMP-donor and whether lysine residue 40 is indeed essential for AMP binding. The adenylyltransferase activity was tested by incubating 1 μg of recombinant protein with α-[^32^P]-ATP. A complex was formed between the wild-type protein and [^32^P]-AMP, which was completely absent for the K40A mutant, confirming that DRB0100 possesses adenylyltransferase activity and therefore belongs to the family of nucleotidyltransferases (Figure 2B). We further tested whether DRB0100 is able to ligate DNA or RNA substrates using NAD^+ ^or ATP as a cofactor. However, we did not detect a ligation product with any conditions used [see Additional file 1 and Additional file 2].

### Purification of a putative 5'-polynucleotide kinase/3'-phosphatase from *D. radiodurans *with an unusual domain architecture

**Figure 4:**
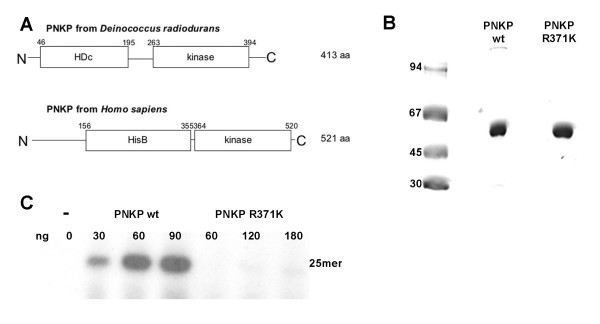
**Purification of a putative PNKP from *D. radiodurans *and analysis of its 5' kinase and 3' phosphatase activities**. A. Scheme of PNKP from *D. radiodurans *and *H. sapiens*. Protein domains are depicted according to predictions based on sequence similarities [36]. Schemes are not drawn to scale. HD, HD domain, kinase, polynucleotide kinase domain, HisB, histidinol phosphatase and related phosphatases domain. B. 3 μg of either *D. radiodurans *PNKP wt or PNKP R371K mutant were loaded onto a 10% SDS-PAGE and subsequently stained with Coomassie Blue R250. Both proteins were purified over 3 columns. Details are described in Methods. C. Titration of the *D. radiodurans *PNKP wt and PNKP R371K mutant to compare their polynucleotide kinase activity on a 5'OH 25 mer deoxyribose oligonucleotide. Different amounts of enzyme were incubated with the DNA substrate and γ-[^32^P]-ATP as described in Methods. [^32^P]-labelled 25 mer was detected by autoradiography.

The PNKP encoded by *D. radiodurans *has a phosphatase-kinase domain architecture similar to the eukaryotic PNKPs. In contrast, the viral T4 PNK has a reverse domain order with the kinase domain at the N-terminus and the phosphatase domain at the C-terminus. Comparison of *D. radiodurans *and human PNKP shows that the bacterial protein is smaller than the human homolog and contains a phosphatase domain belonging to the superfamily of HD phosphohydrolases. The human enzyme contains a distinct phosphatase domain with some similarity to histidinol phosphatase and related phosphatases (Figure 4A).

The gene DRB0098 encoding a putative PNKP was amplified via PCR from genomic *D. radiodurans *DNA. The gene was cloned into a pRSETb vector and arginine 371 was mutated to lysine using mutagenic primers for PCR. Arginine 371 was chosen based on sequence comparisons with the well-characterised T4 PNK. We estimated that it should correspond to arginine 126 in T4 PNK, which is required for polynucleotide kinase activity [20].

Both proteins, DRB0098 wt and DRB0098 R371K were expressed in *E. coli *BL21(DE3) cells with an N-terminal hexa-histidine tag. The proteins were purified over a HisTrap HP™ column, a HiTrap Heparin HP™ column and finally a HiTrap SP HP™ column to apparent homogeneity (Figure 4B).

### Analysis of the *D. radiodurans *PNKP polynucleotide kinase/3'-phosphatase activity

Polynucleotide activity for the *D. radiodurans *PNKP was shown as transfer of ^32^P_i _from γ-[^32^P]-ATP to the 5'OH end of a 25 mer oligodeoxyribonucleotide. The resulting 5'[^32^P]-labelled product was separated from the free γ-[^32^P]-ATP by polyacrylamide gel electrophoresis and detected by autoradiography. The wild-type protein showed clear 5'-polynucleotide kinase activity with an optimal MnCl_2_ concentration of only 0.25 mM. Mutation of arginine 371 to a lysine strongly reduced the enzymatic activity (Figure 4C), confirming that the kinase activity is intrinsic to the C-terminal domain. Furthermore, as *E. coli *does not possess a polynucleotide kinase, a contamination can be excluded.

The 3'-phosphatase activity was analysed as conversion of a non-ligatable DNA nick, which is "blocked" by a 3' PO_4 _moiety, to a normal 3'OH-5'PO_4 _nick that can be subsequently joined by a DNA ligase. Both, D. *radiodurans *LigA and T4 DNA ligase, were able to ligate the blocked substrate if PNKP was present (data not shown). Even though 3'-phosphatase activity has been detected, we cannot conclude whether this activity is intrinsic to the PNKP or not. Samples purified from *E. coli *cells containing only the empty expression vector contained unspecific 3'-phosphatase activity as well and H81A or D82E mutants of DRB0098 did not show any reduced 3'-phosphatase activity, although these two residues represent the conserved HD motif (data not shown). An enzymatic mutant of DRB0098 is required to definitely decide this open question.

## Discussion

DNA ligases are important enzymes acting in DNA replication, recombination and repair. They can be classified by cofactor requirement: those requiring NAD^+ ^and those requiring ATP [21]. For many years it was believed that bacteria possess only NAD^+^-dependent DNA ligases. However, several years ago, it became clear that some bacteria contain an ATP-dependent DNA ligase in addition to their NAD^+^-dependent DNA ligase [22]. The presence of these ligases suggested that prokaryotes, similar to eukaryotes, could have specific DNA ligases that act in DNA repair and recombination.

In this work, we report the identification of LigA, an NAD^+^-dependent DNA ligase, and a second putative ATP-dependent DNA ligase in the radioresistant bacterium *D. radiodurans*. NAD^+^-dependent DNA ligases are highly conserved and it is likely that they are essential for all bacteria [7]. *D. radiodurans *LigA showed strong ligation activity on a nicked DNA substrate in the presence of NAD^+ ^and MnCl_2_, but only a weak activity in the presence of MgCl_2_. This Mn^2+ ^preference is not surprising since it was shown that these ions are present in extremely high levels at the *D. radiodurans *DNA [23] and are essential for γ-radiation resistance [3]. Moreover, several DNA repair enzymes from *D. radiodurans*, such as UV endonuclease β [24] or a family X DNA polymerase with a structure-modulated nuclease activity [25,26], are strongly stimulated by MnCl_2_. The first step in the ligation process is the formation of an adenylated ligase. According to sequence alignment with other NAD^+^-dependent DNA ligases, adenylation of the LigA protein is predicted to occur on lysine 128. Indeed, a mutation of this lysine residue to alanine abolished the ligation as well the adenylation activity.

The product of the *D. radiodurans *gene DRB0100, a diverged homolog of ATP-dependent DNA ligases, contains most of the conserved amino acid residues characteristic of DNA ligases and was shown to be strongly upregulated upon γ-irradation [8]. We could show that this protein possesses adenylyltransferase activity using α-[^32^P]-ATP as a substrate and that the adenylation occurs specifically at the conserved lysine 40. This transfer of radioactivity to the wild-type enzyme, but not to the K40A mutant, indicates a covalent modification of the respective lysine residue as observed for other ligases. This places DRB0100 in the family of nucleotidyltransferases that includes DNA and RNA ligases as well as RNA capping enzymes. As RNA capping is not characterised for prokaryotes, we focussed our work on the possible ligation activity. However, to our knowledge the presence of RNA capping has not been investigated in *D. radiodurans *and can therefore not completely be excluded. Although different cofactors and various buffer conditions as well as different substrates were used, and the hexa-histidine tag was transferred from the N- to the C-terminus of the protein, we were not able to show that DRB0100 is active as a DNA or RNA ligase. Nicked DNA substrates, nicked DNA-RNA hybrids prepared by annealing of a 5' PO_4 _and a 3' OH RNA strand to a template DNA strand, single-stranded RNA and double-stranded DNA with blunt-ends or overhangs were tested (data not shown). In addition, we analysed total *D. radiodurans *extract with or without previous γ-irradiation for DNA ligation activity; however no ATP-dependent ligation activity was detectable, even though NAD^+^-dependent DNA ligation could be easily detected (data not shown). DRB0100 does not contain any conventional DNA binding motif, suggesting that an additional protein is required for recruitment to nicked DNA.

As DRB0100 is part of a putative repair operon DRB0098-DRB0100, we purified the other two proteins to analyse whether the three operon proteins would form a complex capable of DNA ligation. DRB0098 contains a HD-hydrolase family phosphatase domain and a polynucleotide kinase domain and resembles the human repair protein PNKP [27]; DRB0099 is an open reading frame with unknown function and weak similarity to macro domains [8,10]. No DNA ligation was detected with any of these three operon proteins or in combinations thereof; thus, we propose the existence of a yet unidentified additional protein involved in the ligation process of DRB0100. Moreover, it cannot be excluded that DRB0100 ligates only special substrates such as specific DNA sequences or RNA intermediates. Interestingly, in several bacteria genes coding for an ATP-dependent DNA ligase have been identified in operons with Ku-homologs. The Ku proteins might recruit the DNA ligase to DNA strand-breaks as is it the case in mammalian cells [28]. In *D. radiodurans*, however, no Ku-homolog has been identified so far. Another interesting protein that might function in a Ku-like manner is the repair protein PprA from *D. radiodurans*, which has been shown to tether DNA ends and to stimulate ATP- and NAD^+^-dependent DNA ligases [29,30]. The ATP-dependent DNA ligase might function as a backup system to provide additional ligation activity under conditions of high genotoxic stress.

In this work, we furthermore characterised a novel PNKP from *D. radiodurans*, which phosphorylates 5' OH termini. It remains unclear whether it is also able to remove 3' phosphate groups, thus converting "blocked" DNA nicks to ligatable ones.

PNKPs can be divided into two subgroups according to their domain architecture: the T4-like kinase-phosphatase proteins found in viruses with a function in RNA repair, and the eukaryal-type phosphatase-kinase group involved in DNA repair. The PNKP from *D. radiodurans *possesses a domain architecture that corresponds to the eukaryal type. So far, only one bacterial PNKP from *Chlostridium thermocellum *has been described, which in contrast to the *D. radiodurans *PNKP contains a calcineurin-type phosphatase domain. This enzyme has been shown to possess 5'-polynucleotide kinase, 2'3'-phosphatase and adenylyltransferase activity and has been implicated in RNA repair [17]. It remains to be elucidated if *D. radiodurans *PNKP is involved in DNA or RNA repair.

The *D. radiodurans *PNKP possesses an N-terminal phosphatase domain belonging to the HD superfamily. Members of this family are known or predicted phosphohydrolases [18], and a novel subfamily of PNKPs consisting of a 5'-kinase and a 3'-HD phosphohydrolase domain has been proposed based on sequence similarities [8,9,19]. These enzymes have a conserved doublet of HD residues that is likely to be required for enzymatic activity. So far, only one PNKP has been shown to possess a 3'-phosphatase activity residing in the HD domain, but no mutational analysis is available for this enzyme from the bacteriophage RM378 [19]. However, it was shown, that site-directed mutagenesis of the conserved histidine in a cGMP-phosphodiesterase clearly reduced its catalytic activity [31]. We could show that *D. radiodurans *PNKP possesses 5'-polynucleotide kinase activity. However, the 3'-phosphatase activity detected in our assay might result from an unspecific *E. coli *3'-phosphatase. H81A or D82E mutants of *D. radiodurans *PNKP did not show a reduced activity in our 3'-phosphatase assays (data not shown). Regarding the polynucleotide kinase activity, the absence of a 5'-polynucleotide kinase in *E. coli *and the reduced activity of the DRB0098 R371K mutant exclude the possibility of a contamination. In the case of the third protein of the putative repair operon, DRB0099, binding to ADP-ribose was detected and further work has to be done to elucidate whether ADP-ribosylation might play a role in bacterial DNA repair (Blasius, M., and Hübscher, U., unpublished observation).

## Conclusion

*D. radiodurans *possesses a classical NAD^+^-dependent DNA ligase (LigA) that shows a strong preference for Mn(II) as a cofactor. A second predicted ATP-dependent DNA ligase (DRB0100) shows adenylyltransferase activity, but no DNA or RNA ligation could be detected *in vitro*. A predicted 5'-polynucleotide kinase/3'-phosphatase belonging to the same operon was able to convert 5' OH termini to 5' PO_4 _termini, thus preparing DNA ends for ligation. In conclusion, *D. radiodurans *PNKP and LigA are able to heal and ligate DNA nicks. It remains to be assessed whether they play any role in DNA repair or RNA repair *in vivo*. Also the function of DRB0100 remains to be elucidated and further proteomic and genomic approaches might give more insight into these unsolved questions.

## Methods

### Bacterial strains and media

*E. coli *DH5α cells were used for cloning and plasmid preparation (Invitrogen). Recombinant proteins were produced in *E. coli *BL21(DE3) (Novagen). *E. coli *cells were grown in LB medium supplemented with 100 μg/ml ampicillin where required.

### Enzymes and reagents

Oligonucleotides synthesis and DNA sequencing were performed by Microsynth. DNA fragments and plasmids were purified with kits from Qiagen. All chemicals used were purchased from Sigma-Aldrich. Immunoblots during protein purifications were done using Tetra-His antibody (Qiagen).

### Molecular cloning

Genomic DNA was isolated from *D. radiodurans *R1 type strain as described previously [32] and used as a template for PCR amplification of the different genes. PCR reaction mixtures (50 μl) contained 1X HF buffer (Finnzymes), 200 μM of each dNTP, 400 nM of each forward and reverse primer, 3% DMSO and 2 units of Phusion™ High-Fidelity DNA Polymerase (Finnzymes). Cycling protocols were designed according to the supplier's recommendations and annealing temperatures were determined using the Tm calculator provided by Finnzymes. PCR products were digested with BamHI and ligated into the pRSETb vector (Invitrogen) using T4 DNA ligase (Fermentas). For site-directed mutageneses the plasmid containing the corresponding wild-type gene was used as a template, the annealing temperature was set to 55°C, cycle number was reduced to 12–16, and the PCR product was digested with DpnI to remove the template plasmid. The mutated PCR product was then transformed into DH5α cells, plasmids were isolated and all constructs were verified by sequencing. PCR primer sequences can be found in Table 1.

### Expression and purification of recombinant proteins

Cultures of *E. coli *BL21(DE3) cells transformed with the respective expression plasmid were grown in LB medium supplemented with ampicillin at 37°C to an OD_600 nm _of 0.4–0.8, then IPTG was added to 1 mM final concentration and cells were further incubated for 2–4 h at 37°C. Cells were pelleted by centrifugation (4°C, 4,700 g, 30 minutes) in a Sorvall H6000A rotor. All protein purification steps were performed at 4°C or on ice. Cell pellets were resuspended in 30 ml of buffer N (500 mM NaCl, 30 mM phosphate buffer, pH 7.5, 10 mM Tris-HCl, pH 7.5, 10 mM imidazole, and 1 mM PMSF) and lysed with a French press. To ensure complete lysis, cells were in addition sonicated (2 minutes, 40% duty cycle, Branson Sonifier^® ^Cell disruptor B15). The lysate was centrifuged (4°C, 43,000 g, 30 minutes) in a Sorvall SS-34 rotor and the supernatant was loaded onto a 1 ml HisTrap HP™ column (GE Healthcare) using an ÄKTApurifier™ (GE Healthcare). The column was washed with buffer N containing 50 mM NaCl and 50 mM imidazole and protein was eluted with 50 mM NaCl and 300 mM imidazole. Protein was pooled according to a Coomassie Blue R250 stained SDS-PAGE and loaded onto the next column equilibrated in buffer A (40 mM Tris-HCl, pH 7.5, 50 mM NaCl, 15% (v/v) glycerol, 1 mM EDTA, and 1 mM 2-mercaptoethanol). For LigA wt and K128A mutant the protein was loaded onto a 1 ml Heparin HP™ column and eluted with a gradient from 50 to 1000 mM NaCl in buffer A. LigA eluted at 350 mM NaCl. Protein was pooled, diluted to 50 mM NaCl with buffer A without NaCl and loaded onto a HiTrap Q HP™ column. Elution was done with a gradient from 50–1000 mM NaCl. Protein eluted at 400 mM NaCl. Fractions that contained nearly homogenous LigA protein as judged by SDS-PAGE were pooled, dialysed to buffer S (20 mM Tris-HCl, pH 7.5, 25% (v/v) glycerol, 50 mM NaCl, 1 mM 2-mercaptoethanol, and 0.5 mM EDTA), and stored at -80°C.

For DRB0100 wt and K40A mutant the pool obtained from the HisTrap HP™ column was loaded onto a 1 ml HiTrap SP HP™ column equilibrated with buffer A. DRB0100 was retrieved in the flow-through, which was diluted to 25 mM NaCl and loaded onto a HiTrap Q HP™ column. Elution was done using a gradient from 25–1000 mM NaCl and DRB0100 protein eluted at 50 mM NaCl. The protein pool was dialysed to buffer S and stored at -80°C.

The HisTrap HP™ pools of DRB0098 wt and R371K mutant were loaded onto a 1 ml Heparin column and eluted as described for LigA. Protein was pooled, diluted to 25 mM NaCl and loaded onto a 1 ml SP HP™ column. Protein was eluted at 50 mM NaCl, tested for purity as described above, pooled and dialysed to buffer S for storage at -80°C. Protein concentrations were determined using bovine serum albumin standards and a BioRad protein assay.

### Adenylyltransferase activity assays

For DRB0100, reaction mixture (20 μl) containing 50 mM Tris-HCl, pH 7.5, 5 mM dithiothreitol, 5 mM MgCl_2_, 1.25 μM α-[^32^P]-ATP and the indicated amounts of protein were incubated for 15 minutes at 30°C. 20 μl of 2X Laemmli buffer were added, samples were heated for 5 minutes at 95°C and products were separated on a 12% standard SDS-PAGE. The intermediates were detected by autoradiography and the gel was stained by Coomassie Blue R250 to visualize the molecular weight markers. For LigA, the reaction mixture (10 μl) contained 50 mM Tris-HCl, pH 6.8, 5 mM dithiothreitol, 1 mM MnCl_2_, 1 μg of protein and 0.1 μM [^32^P]-NAD^+^. The reaction was incubated for 15 minutes at 30°C, stopped with 10 μl of 2X Laemmli buffer, heated for 5 minutes at 95°C and loaded onto a 10% SDS-PAGE. Prestained markers were loaded to compare protein sizes. Free [^32^P]-NAD^+^ and ligase-AMP complexes were visualized by autoradiography.

### Preparation of DNA substrates

The DNA substrate used to measure the ligation activity on a double-stranded substrate carrying a single-strand nick was prepared as described [33], the 19 nucleotide DNA strand was phosphorylated using γ-[^32^P]-ATP and T4 polynucleotide kinase (New England Biolabs). Free γ-[^32^P]-ATP was removed on a MicroSpin™ G-25 column (GE Healthcare). The sequences are presented in Table 3.

**Table 3:** Oligonucleotide sequences used to prepare DNA substrates for the enzyme assays performed in this study

**Oligonucleotide**	**Length (nt)**	**Sequence (5'-3')**
RNA-5' nick	19	CAGCAGCAAAUGAAAAAUC
DNA-5' nick	19	CAGCAGCAAATGAAAAATC
RNA-3' nick	25	CCUGCAACAGUGCCACGCUGAGAGC
DNA-3' nick	25	CCTGCAACAGTGCCACGCTGAGAGC
DNA-3'P nick	25	CCTGCAACAGTGCCACGCTGAGAGC-P
DNA-opposite	46	AGATTTTTCATTTGCTGCTGGCTCTCAGCGTGGCACTGTTGCAGGC
Kinase-DNA	25	GCTTTCCGAGTACCGGGGTCTTCCG

All oligonucleotides were PAGE purified and labelled as described in the Methods section. P stands for a 3' phosphate group.

### Ligation assays

The 5'-[^32^P]-labelled DNA substrate was incubated for 30 minutes with the indicated amounts of recombinant protein at 30°C. Reactions were performed in a final volume of 10 μl containing 50 fmol of 5'-[^32^P]-labelled DNA, 50 mM Tris-HCl, pH 6.8, 1 mM MnCl_2_, 5 mM dithiothreitol and 1 mM ATP or 5 μM NAD^+ ^unless otherwise mentioned. The reactions were stopped by adding 10 μl of loading buffer (95% formamide, 20 mM EDTA, 0.05% bromphenol blue, 0.05% xylene cyanol), heated for 5 minutes at 95°C and products were separated on a 15% denaturing polyacrylamide gel containing 8 M urea and 15% formamide. After autoradiography, the signals were quantified on a PhosphorImager using the ImageQuant software (Molecular Dynamics). Ligation was quantified by calculating the product/(product+substrate) ratio thus allowing a correction for loading errors. To determine the *K*_Mnicked DNA _value of the *D. radiodurans *NAD^+^-dependent DNA ligase, the reactions contained 50 mM Tris-HCl, pH 6.8, 5 mM dithiothreitol, 1 mM MnCl_2_, 5 μM NAD^+^, 10–150 fmol of [^32^P]-labelled nicked DNA substrate and 2 ng of enzyme. The reactions were incubated for 15 minutes at 30°C. The ligated products were quantified by PhosphorImager and *K*_Mnicked DNA _was calculated by Lineweaver-Burk plotting as a mean of 3 independent experiments.

### Polynucleotide kinase assays

The indicated amounts of recombinant protein were incubated with 1 pmol of DNA substrate (kinase-DNA in Table 3) in a volume of 10 μl containing 50 mM Tris-HCl, pH 7.5, 0.25 mM MnCl_2_, 5 mM dithiothreitol and 0.25 μCi of γ-[^32^P]-ATP (GE Healthcare) for 30 minutes at 30°C. Reactions were stopped with 10 μl of loading buffer, heated for 5 minutes at 95°C and separated on a 15% denaturing polyacrylamide gel containing 8 M urea and 15% formamide. Signals of ^32^P-DNA were visualized by autoradiography.

## Authors' contributions

MB performed most of the experiments, did most of the cloning and protein purifications, analysed the data and wrote the manuscript. RB helped characterising the NAD^+^-dependent DNA ligase. IS performed part of the cloning and protein purification. MB, IS and UH conceived of the study. UH helped analysing the data and preparing the manuscript. All authors read and approved the final version of the manuscript.

## Supplementary Material

Additional file 1 Factors tested to detect DNA ligation activity of the DRB0100 gene product. This table lists the various buffer conditions and proteins tested in DNA ligation assays for the DRB0100 gene product. Click here for file

Additional file 2 Ligation substrates tested to detect DNA ligation activity of the DRB0100 gene product. This table shows the sequences of the different oligonucleotides that were used to prepare ligation substrates for the DRB0100 gene product. Click here for file
