# Supplementary material for: Enzymes involved in DNA ligation and end-healing in the radioresistant bacterium Deinococcus radiodurans
Source: BMC Mol Biol. 2007 Aug 16;8:69. doi: 10.1186/1471-2199-8-69 (PMC1997131; doi:10.1186/1471-2199-8-69)
Supplement: Additional file 1 — Factors tested to detect DNA ligation activity of the DRB0100 gene product. This table lists the various buffer conditions and proteins tested in DNA ligation assays for the DRB0100 gene product. [file 1471-2199-8-69-S1.pdf]

Additional file 1. Factors tested to detect DNA ligation activity of the DRB0100 gene product

Factor	***Details***
pH	5.5- 8.5
AMP donor	ATP or NAD+
divalent cation	Mn^{2+}, Mg^{2+}, Co^{2+} or Ni^{2+}
Proteins added	***Origin***
D. radiodurans PprA	Gift from I. Narumi
D. radiodurans LigA	this work
D. radiodurans PNKP	this work
D. radiodurans SSB	Gift from M. Cox
D. radiodurans RecA	Gift from M. Cox
D. radiodurans DdrA	Gift from M. Cox
Bovine Serum Albumin	New England Biolabs
