# Supplementary material for: Enzymes involved in DNA ligation and end-healing in the radioresistant bacterium Deinococcus radiodurans
Source: BMC Mol Biol. 2007 Aug 16;8:69. doi: 10.1186/1471-2199-8-69 (PMC1997131; doi:10.1186/1471-2199-8-69)
Supplement: Additional file 2 — Ligation substrates tested to detect DNA ligation activity of the DRB0100 gene product. This table shows the sequences of the different oligonucleotides that were used to prepare ligation substrates for the DRB0100 gene product. [file 1471-2199-8-69-S2.pdf]

Additional file 2. Ligation substrates tested to detect DNA ligation activity of the DRB0100 gene product

Ligation substrates prepared by annealing of the following oligonucleotides (* indicates ^{32}P label)
5'-*CCUGCAACAGUGCCACGCUGAGAGC-3'
5'-CCUGCAACAGUGCCACGCUGAGAGC-3'<br>5'-*CAGCAGCAAAUGAAAAAUC-3'<br>5'-AGATTTTTCATTTGCTGCTGGCTCTCAGCGTGGCACTGTTGCAGGC-3'
5'-CCTGCAACAGTGCCACGCTGAGAGC-3'<br>5'-*CAGCAGCAAAUGAAAAAUC-3'<br>5'-AGATTTTTCATTTGCTGCTGGCTCTCAGCGTGGCACTGTTGCAGGC-3'
5'-CCUGCAACAGUGCCACGCUGAGAGC-3'<br>5'-*CAGCAGCAAATGAAAAATC-3'<br>5'-AGATTTTTCATTTGCTGCTGGCTCTCAGCGTGGCACTGTTGCAGGC-3'
5'-*CTAGGTTAGCATGACCGCTG-3'<br>5'-GTCATGCTAACCTAGCAGCG-3'

Ligation substrates tested to detect DNA ligation activity of the DRB0100 gene product. In addition, various blunt-ended DNA substrates have been tested. The substrates have also been used for electromobility shift experiments, but no binding of the DRB0100 gene was detectable.
